# Live ascaris in urinary bladder: a case report

**DOI:** 10.1186/s13256-021-03045-4

**Published:** 2021-09-30

**Authors:** Gashaw Mesele, Zelalem Mengistu

**Affiliations:** 1 Department of Surgery, University of Gondar, Gondar, Ethiopia; 2 Department of Obstetrics and Gynecology, University of Gondar, Gondar, Ethiopia

**Keywords:** *Ascaris lumbricoides*, Urinary helminthiasis, Urinary ascariasis, Urinary parasites

## Abstract

**Introduction:**

Ascaris in urinary bladder is an extremely rare phenomenon. It may occur after fistula formation between urinary and gastrointestinal tract or by retrograde migration of adult worm, and is associated with complications.

**Case presentation:**

A 47-year-old Amhara woman from rural northwest Ethiopia presented with a complaint of difficulty to fully evacuate her bladder of 1 year duration. Ultrasonography showed thickened bladder wall with echo debris. There were also thickened bowel and fluid-filled loops of intestine adjacent to urinary bladder. On cystoscopy examination, there was live ascaris swimming inside the bladder. Enterovesical fistula was entertained and explorative laparotomy performed. Findings confirmed presence of iliovesical fistula. The fistula was divided and the continuity of the intestine restored. The inflammatory mass was subjected to histopathology study and turned out to be benign inflammatory reaction. She was also given antihelminthics. Postoperatively, her course was uneventful, and she was discharged cured.

**Conclusion:**

Though it is extremely rare to have urinary symptoms from ascariasis, it is important to have a high index of suspicion for all possibilities.

## Introduction

Soil-transmitted helminth (STH) infections like ascariasis are a public health problem in developing countries where adequate method of disposal of human excreta is not available and affect more than one billion people [1, 2].

People infected with ascaris are often asymptomatic. When it becomes symptomatic, ascariasis presents with myriad of clinical pictures depending on intensity of the infection, the nutritional and immunologic status of the host, and the possible complications that may arise. The sites of involvement are mainly lung during larval migration and intestine after full growth of the helminth. Invasion of the biliary ducts and the liver parenchyma may occur. The adult worm has been reported also in pleural cavity, pancreas, peritoneal cavity, lacrimal duct, middle ear, and femoral artery [3, 4].

Urinary complications of ascaris are extremely rare [5, 6]. There are very few case reports that are assumed to result from either fistulus communication between the genitourinary system and alimentary canal or due to transurethral access of the worm to the genitourinary system [7]. None of the reports proves the proposed theories; however, in our case, enterovesical fistula formation was clearly demonstrated during laparotomy, which consolidates the theory of fistula formation between the bowel and the urinary system as the reason for finding ascaris in urinary system.

## Case presentation

A 47-year-old para five (all alive and healthy) Amhara woman from rural northwest Ethiopia without remarkable past medical history presented with a complaint of difficulty to fully evacuate her bladder of 1 year duration associated with protruding mass per vagina. Later, she reported voiding was only possible after reduction of the prolapsed organs. She also reported frequent treatment for urinary tract infection with ciprofloxacillin and norfloxacillin in the last 1 year at a nearby health facility. She has no history of smoking or alcohol consumption. She is a farmer living with her husband and five of her children, and is socially active.

On examination at admission, she was chronically sick looking with normal blood pressure (110/70 mmHg), pulse rate (88 beats per minute), and temperature (36.5 ℃). She had pink conjunctiva, nonicteric sclera, no anterior neck mass, clear and resonant chest, quiet precordium, S1 and S2 heart sound well heard, no murmur or gallop, and flat abdomen without tenderness or palpable mass. On genitourinary examination, she had protruded mass per vagina with uterine cervix as the leading point 6 cm below the hymenal ring. Otherwise, there were no pertinent positive findings on other parts of her body. On neurologic examination, she had intact sensory and motor function.

**Fig. 1:**
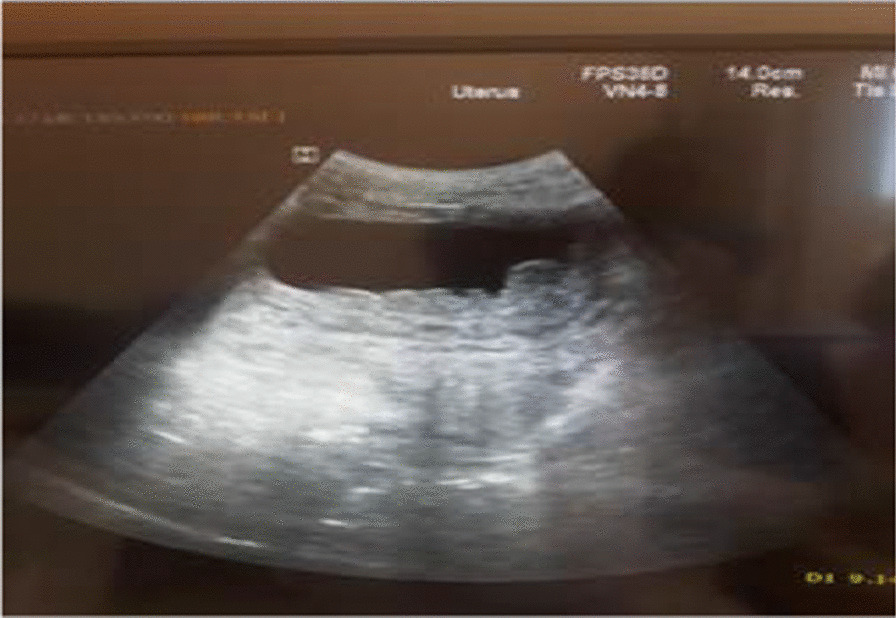
Thickened bowel and fluid-filled loops of intestine adjacent to urinary bladder

On her laboratory tests, her hematocrit was 38% with normal renal [blood urea nitrogen (BUN) = 20, creatinin = 0.6] and liver functions (serum glutamic oxaloacetic transaminase (SGOT) = 22, serum glutamic pyruvic transaminase (SGPT) = 37); human immunodeficiency virus (HIV) test was negative; urine test showed white blood cell (WBC) count more than 10/high-power field (HPF) and there were no ova or parasites on stool examination. On ultrasonography, there was thickened bladder wall with echo debris. There were also thickened bowel and fluid-filled loops of intestine adjacent to urinary bladder (Fig. 1). No radiographic or other scanning was done.

**Fig. 2:**
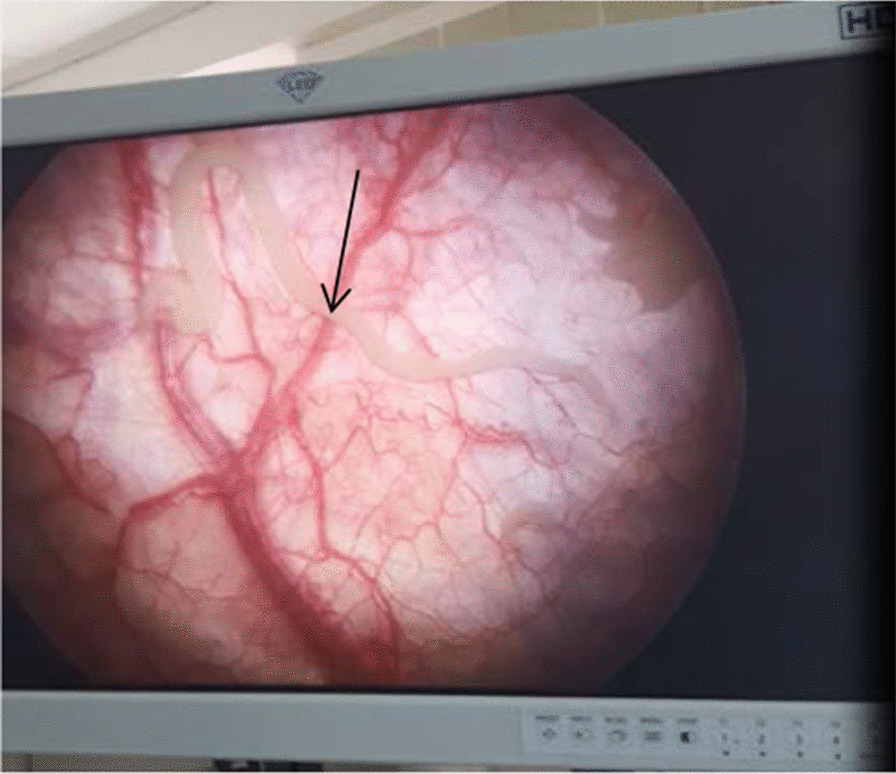
Live ascaris swimming inside the bladder. Arrow shows swimming Ascaris

**Fig. 3:**
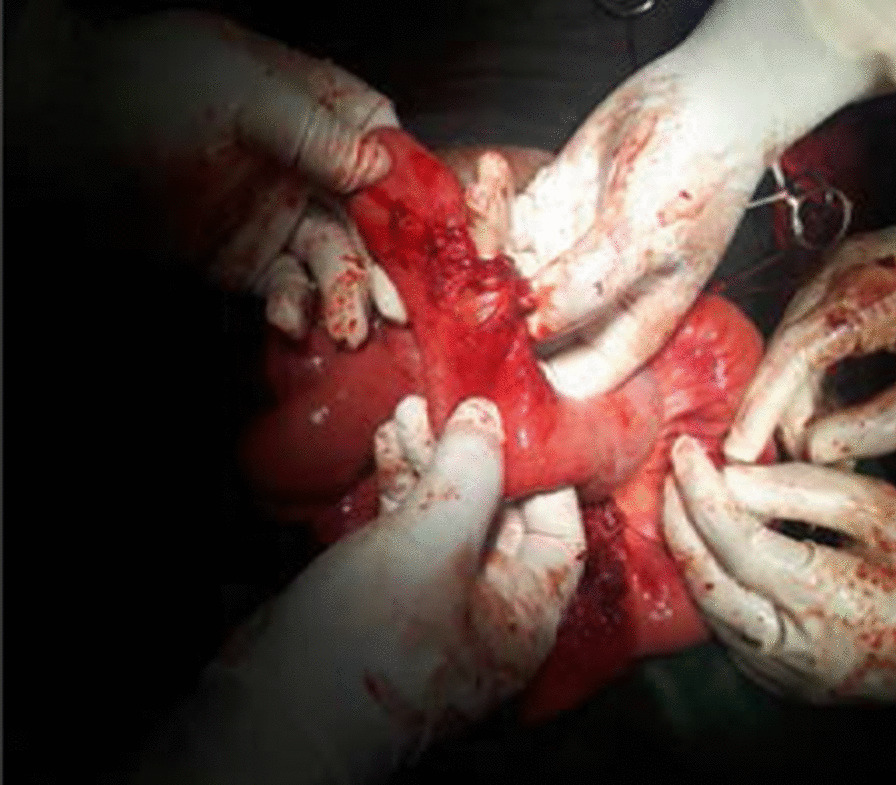
Perforation of ileum

To investigate the bladder mass, we did cystoscopy where we were able to see live ascaris swimming inside the bladder (Fig. 2). It also revealed that there was bladder mucosal edema everywhere and broad-based non-ulcerated mass arising from the dome. Enterovesical fistula was entertained and explorative laparotomy performed. Findings confirmed presence of iliovesical fistula (Fig. 3). Mass was of inflammatory origin, and there were no evidence of chronic inflammatory bowel disease of the affected small bowel. The fistula was divided and the continuity of the intestine restored. The inflammatory mass was subjected to histopathology study and turned out to be benign inflammatory reaction. Two weeks later, the pelvic organ prolapse was managed with hysterectomy and right sacrospinous ligament suspension and bilateral vaginal repair. She was also given albendazole 400 mg po stat and was discharged cured after 3 days of pelvic organ prolapse surgery. She had follow-up after 6 months and was doing fine.

## Discussion

We have presented a women who presented to our facility with difficulty emptying her bladder and protruding mass per vagina of 1 year duration. Ultrasonography was done and showed thickened bladder wall with echo debris and thickened bowel with fluid-filled loops of intestine adjacent to urinary bladder. Thus, cystoscopy was done, which revealed live ascaris swimming in bladder.

Urinary ascariasis (UA) is an extremely rare phenomenon [5, 6]. UA is assumed to result from either fistula formation between the urinary system and alimentary canal or due to transurethral access of the worm into the urinary system [7]. The most likely explanation for transurethral access might be a worm potentially forced to migrate and exit per anus by deworming therapy, and subsequently moving transperineally to ascend into the urethra and bladder [6]. In our case, the ascaris accessed the bladder through the iliovesical fistula as demonstrated during laparotomy, which supports the theory that fistula formation is a reason for urinary bladder ascariasis, which makes our case peculiar since it was not demonstrated in other case reports so far. There was no other pathology identified in the rest of the bowel; this makes other concomitant systemic pathologies like Crohn’s disease, tuberculosis, or malignancy an unlikely cause for the fistula formation. The likely explanation is intestinal perforation due to ascaris, which subsequently elicited inflammation in the bladder, resulting in fistulous communication between bladder and ileum.

## Conclusion

Though it is extremely rare to have urinary symptoms from ascariasis, it is important to have a high index of suspicion for all possibilities, particularly when other possibilities are absent and the patient is not responding to treatments. This case report demonstrated the consequence of ascariasis, emphasizing the need for regular deworming for vulnerable sections of the community, such as farmers.

## Abbreviations

STH: Soil-transmitted helminth
UA: Urinary ascariasis
